# Study on the impacts of chemical and green synthesized (*Leucas aspera* and oxy-cyclodextrin complex) dietary zinc oxide nanoparticles in Nile tilapia (*Oreochromis niloticus*)

**DOI:** 10.1007/s11356-020-11992-6

**Published:** 2021-01-06

**Authors:** Amitha Kurian, Preetham Elumalai

**Affiliations:** School of Ocean Science and Technology, Kerala University of Fisheries and Ocean Studies, Panangad, Kochi, Kerala 682 506 India

**Keywords:** ZnO nanoparticle, Green synthesis, Chemical synthesis, Toxicity, Histology, Immunity, Bioaccumulation

## Abstract

The present study was designed to evaluate the health effects of dietary nanozinc prepared by two methods: conventional chemical method and green method. The parameters evaluated were the extent of bioaccumulation, antioxidant status, histological, immunological changes and DNA damage in Nile tilapia fed nanozinc feed. Zinc oxide nanoparticles were first prepared by green and chemical methods. Before feed preparation, the in vitro antioxidant activity and antibacterial activity of both types of nanoparticle solutions were tested and the results revealed enhanced activities in green synthesized ZnO NP solution. After the acclimatization period, 420 Nile tilapias were distributed randomly into 21 glass tanks with 20 fish per tank in triplicates. Fish were fed control diet without any ZnO NP and (i) GT1—green synthesized ZnO NP diet at 100 mg/kg, (ii) CT1—chemically synthesized ZnO NP diet at 100 mg/kg, (iii) GT2—green synthesized ZnO NP diet at 200 mg/kg, (iv) CT2—chemically synthesized ZnO NP diet at 200 mg/kg, (v) GT3—green synthesized ZnO NP diet at 400 mg/kg and (vi) CT3—chemically synthesized ZnO NP diet at 400 mg/kg for 60 days. After 60 days, gill and liver samples were collected for analysing oxidative stress, histopathological alterations and bioaccumulation of zinc, whereas serum samples were collected for evaluating immune response. The results revealed that the GT3 diet significantly (*P* < 0.05) enhanced the level of antioxidant enzymes (CAT, SOD, GPx, GR and GSH) than dietary nanozinc prepared by the chemical method. Similarly, the innate immunological parameters were significantly (*P* < 0.05) augmented in fish fed GT3 diet. Comparative histological study of liver and gill tissues revealed normal architecture in the tissues of fish fed green synthesized NP–enriched feed, whereas the tissues of fish fed chemically synthesized NP feed exhibited histological alterations. Bioaccumulation of zinc was more in the liver followed by the muscle and least in the gills and DNA damage was more evident in fish fed chemically synthesized ZnO NP–enriched feed. In conclusion, the results suggest that the inclusion of 400 mg/kg GT3 diet in fish diet enhanced the level of antioxidant enzymes, boosted immune response and did not cause histological damage to organs, and therefore, GT3 nanofeed can be recommended for fish health improvement.

## Introduction

Nanotechnology offers incredible applications in almost all spheres of life due to which this branch of science has gained popularity and attention swiftly (Bhattacharya and Mukherjee 2008). Wide application of nanoparticles can induce serious threats to the environment which is why it is important to study the hazards of nanoparticles on the aquatic system (Kumar et al. 2010). One such widely used nanoparticle is zinc oxide. Zinc oxide nanoparticles (ZnO NPs) are commercially utilized (Amornpitoksuk et al. 2011). They have promising health potential like antioxidant (Das et al. 2013), antimicrobial (Premanathan et al. 2011) and anticancer (Li et al. 2010) activities. Plant-mediated synthesis of nanoparticles, also known as green synthesis, is an eco-friendly approach that has reduced the dangers of chemical methods of synthesis to an extent (Sundrarajan et al. 2015). When ZnO NPs come in contact with the water body, they induce oxidative stress to the aquatic animals (Connolly et al. 2016). But no information is available on the toxic effects of dietary ZnO NPs. In this context, we aim to compare the health effects of bio inspired green ZnO NPs and chemically synthesized ZnO NP–enriched feed on Nile tilapia (*Oreochromis niloticus*). Nile tilapia is one of the most popular farmed fish species that has a good market value, can tolerate stress and is considered as an ideal organism for ecotoxicological studies (Abarike et al. 2019; Hasan Kaya et al. 2015). For green synthesis of ZnO NPs, *Leucas aspera* aqueous extract and oxy-cyclodextrin complex (oxy-CD complex) are used. Oxy-CD complex is a water-soluble compound of oxyresveratrol. It is prepared as an inclusion complex based on the method of Nasapon et al. (2010) Oxyresveratrol is a polyphenol, mainly 1, 4′, 3, 5′-tetra hydroxy stilbene. Cyclodextrin forms an inclusion complex by improving the physicochemical properties with other guest molecules without altering the original structure (Venuti et al. 2014). *Leucas aspera* (Willd.) Linn. (Family: *Lamiaceae*) also known as ‘*thumbai*’ is distributed throughout India and is traditionally used as an antipyretic and insecticide (Suganya et al. 2014). *L. aspera* contains medicinally active compounds such as triterpenoids, diterpenes, oleanolic acid, ursolic acid and b-sitosterol, sterols, glucoside, phenolic compounds (4-(24-hydroxy-1-oxo-5-n-propyltetracosanyl)-phenol) and hence is pharmacologically relevant. Our previous study has reported that the inclusion of plants in the diet of Nile tilapia improves immunity (Musthafa et al. 2018) and our recent study reported that *L. aspera* in the diet of Nile tilapia enhances immunity and disease resistance against *Aeromonas hydrophila* (Kurian et al. 2019). There are limited studies in Nile tilapia on the effects of nanofeed on bioaccumulation, oxidative stress response, DNA damage and immunological effects. Hence this study investigates and compares in depth the impacts of green and chemically synthesized nanozinc feed on the health status of Nile tilapia. The outcome of this study can be taken into consideration to decide whether nanozinc feed can be recommended as a safe alternative to conventional fish feeds.

## Materials and Methods

### Preparation of *Leucas aspera* extract

*L. aspera* plants were grown in the campus of Kerala University of Fisheries and Ocean Studies. Fresh *L. aspera* plants were collected from the campus in April 2019 and the aerial parts were rinsed completely in distilled water, chopped and shade dried for around 10 days and ground into fine powder according to Suganya et al. (2014). Five grams of plant powder was added into a conical flask containing 50 ml distilled water and boiled for 20 min. The aqueous extract was filtered using Whatman No.1 filter paper and stored at 4 °C.

### Oxy-cyclodextrin complex

Oxy-cyclodextrin was a kind gift from Dr. Sreeja lakshmi, DHR Research Scientist, KUFOS, Kerala, India.

### Green synthesis and chemical synthesis of zinc oxide nanoparticles

The synthesis of zinc oxide nanoparticles was based on the co-precipitation method described by Singh et al. (2011). Starting materials used were zinc acetate dihydrate (Zn (CH_3_COO)_2_ 2H_2_O; Merck) and sodium hydroxide (NaOH; Merck). A total of 2 M zinc acetate in 50 ml distilled water was prepared under constant stirring. To this, 50 ml of 2 M NaOH was added after complete dissolution of zinc acetate. The mixture was kept in a magnetic stirrer for 2 h. The white precipitate formed was filtered and washed with distilled water and ethanol (Merck) to remove impurities. The purified precipitate was kept in the oven at 60 °C for overnight drying and the resultant solid white powder was kept for calcination in a muffle furnace at 300 °C for 3 h. For green synthesis of zinc oxide nanoparticles, 5 ml of plant extract, 5 ml of oxyresveratrol and 50 ml of 2 M NaOH were added to 50 ml of 2 M zinc acetate solution. The off-white precipitate formed was filtered and washed with distilled water and ethanol to remove impurities. The purified precipitate was kept in the oven at 60 °C for overnight drying and the resultant solid off-white powder was kept for calcination in the muffle furnace at 300 °C for 3 h.

### Characterization of nanoparticles

For understanding various physicochemical features, nanoparticles were characterized by UV-Vis spectroscopy analysis (UV-1800 Shimadzu, Japan), XRD (Bruker D8 advance, Germany), FTIR (IRaffinity 1, Shimadzu, Japan), Zeta potential (Malvern Zetasizer, UK), SEM (EVO 18 Carl Zeiss, Germany) and TEM (FEI-Tecnai G2 20, USA)

### In vitro antioxidant activity

#### DPPH free radical scavenging assay

DPPH free radical scavenging activity was measured by the method of Das et al. (2013) with modification. Various concentrations of ZnO NP solution (25, 75, 100, 200, 400, 600 and 800 μg/ml) in methanol (4 ml) was mixed with 1 ml of freshly prepared DPPH (HiMedia; 0.1 mM in 100% methanol; Merck), the mixture was shaken vigorously and incubated for 30 min at room temperature in the dark. The absorbance was measured at 517 nm against methanol blank in a UV-Vis spectrophotometer (UV-1800 Shimadzu, Japan). DPPH scavenging activity is calculated by the following equation

Scavenging activity (%) = 100 × (*A*_c_ − *A*_s_)/*A*_c_

*A*_c_ and *A*_s_ are absorbance of control and sample respectively.

#### Reducing power assay

The reducing power was determined by Oyaizu’s method (1986) with minor modifications. Different concentrations of 1 ml of ZnO NP solution (25, 75, 100, 200, 400, 600 and 800 μg/ml) was mixed with 2.5 ml of phosphate buffer (0.2 M, Ph 6.6) and 2.5 ml of potassium ferricyanide (HiMedia) and kept at 50 °C for 20 min in a water bath. 2.5 ml of 10% trichloroacetic acid (TCA; Merck) was added to terminate the reaction and centrifuged at 4000 rpm for 5 min. 2.5 ml of the upper layer was diluted with 2.5 ml of distilled water and 0.5 ml of 0.1% ferric chloride (HiMedia) was added. The absorbance was measured at 700 nm in a UV-Vis spectrophotometer (UV-1800 Shimadzu, Japan). Reducing power was represented in the form of optical density (OD). The higher the OD, the higher the reducing power.

### Antibacterial activity

*Aeromonas hydrophila* (*A. hydrophila*) and *Streptococcus agalactiae* (*S. agalactiae*) obtained from CUSAT (Cochin University of Science and Technology), Kochi, Kerala, was sub-cultured for 24 h at 37 °C with agitation to mid-log growth phase and colonies were confirmed as *Aeromonas* and *Streptococcus* using *Aeromonas* special media (HiMedia) and *S. agalactiae* selective agar base prior to use. Antibacterial activity of ZnO NPs against *Aeromonas hydrophila* (*A. hydrophila*) and *Streptococcus agalactiae* (*S. agalactiae*) were tested using the Kirby-Bauer et al. (1966) disc diffusion method. Bacteria strains were spread uniformly on the nutrient agar plates using sterile cotton swabs. Filter paper discs were placed on the agar plates and were loaded with different concentrations of both types of ZnO NPs (25, 50 and 75 μg/ml) and incubated at 37 °C for 24 h. The zone of inhibition was observed after 24 h of incubation.

### Fish and experimental design

Six hundred Nile tilapia were purchased from a fish farm in Alleppey district, Kerala, and were transported to the laboratory. Fish were acclimatized for 14 days in a 1000-L aerated fibre tank and were fed commercial feed (No 9951, CPF company, India; protein content—33.8 g kg^−1^ dry matter basis, lipid content—6.44 8 g kg^−1^ dry matter basis). Then, 5 fish were randomly caught to check the health status by examining the gills and internal organs observation under a light microscope. After the acclimatization period, 420 fish were distributed randomly into 21 glass tanks (150 L; 20 fish per tank; average weight = 20.11 ± 0.14 g) with three replicates per treatment. Throughout the experiment, water quality parameters were monitored daily at 9.30 a.m. and 5.30 p.m. and parameters like pH, temperature, nitrite level, ammonia and dissolved oxygen were tested prior to the study. The pH and temperature were determined by conventional methods whereas dissolved oxygen was measured using manganese oxidation capacity. An API freshwater master test kit was used for checking ammonia and nitrite. Water was changed daily up to 50% to regulate these parameters: water temperature 28 ± 2 °C, pH 7.7 ± 0.24 and dissolved oxygen 5.1 ± 0.31 mg/L. The following diets were fed to fish for 60 days.

(i) Control tank—Not exposed to ZnO NPs, fed with control diet

(ii) GT1—Fish that received green synthesized ZnO NPs at concentration 100 mg/kg

(iii) CT1—Fish that received chemically synthesized ZnO NPs at concentration 100 mg/kg

(iv) GT2—Fish that received green synthesized ZnO NPs at concentration 200 mg/kg

(v) CT2—Fish that received chemically synthesized ZnO NPs at concentration 200 mg/kg

(vi) GT3—Fish that received green synthesized ZnO NPs at concentration 400 mg/kg

(vii) CT3—Fish that received chemically synthesized ZnO NPs at concentration 400 mg/kg

To prepare the nanozinc-enriched diets, green synthesized and chemically synthesized ZnO NP powders were weighed (GT1-100 mg, GT2—200 mg, GT3—400 mg; CT1—100 mg, CT2—200 mg, CT3—400 mg) and dispersed into vegetable oil (20 ml) in separate containers. The doses were chosen based on environmentally relevant concentrations described by Anu et al. (2014). To each of the containers, conventional diet was added and mixed properly to prepare different treatment diets. Similarly, control diet was prepared by mixing the same volume of vegetable oil per kilogramme. The prepared diets were left open in the containers for the oil to get absorbed into the feeds. After completion of drying, the feeds were stored in a cool and dry place. Fish in each replication were fed ad libitum twice a day at 9:00 a.m. and 5:00 p.m. for 60 days. The remaining feed and metabolic wastes were siphoned out daily and renewed water in each tank.

Fish were stopped giving feed 24 h before sample collection. At the end of the feeding trial, four fish were randomly caught from each tank and were anaesthetized with clove oil (5 ml L^−1^), followed by blood collection from the caudal vein with a 1 ml plastic syringe sprayed with heparin (HiMedia) and stored at 4 °C until the next day. Serum was prepared as described by Doan et al. (2016). Using a 1-ml syringe, blood was collected from the caudal vein. The collected blood samples were immediately transferred into Eppendorf tubes and were allowed to clot for 1 h at room temperature and 4 h at 4 °C. The samples were centrifuged at 1500×*g*, for 5 min, at 4 °C in a cooling centrifuge (Heraeus Thermo Scientific Megafuge 8R, Germany). The serum was finally collected and stored at − 20 °C until assay. At the end of the feeding period, gills and liver from each group were collected, weighed, homogenized in potassium phosphate buffer (50 mM, pH 7.0) and centrifuged at 10,000 rpm for 10 min at 4 °C. The supernatant collected was used for determining the antioxidant enzyme activities. The concentration of protein in tissue homogenates was measured according to the method of Lowry et al. (1951), using bovine serum albumin (HiMedia) as standard.

### Enzymatic antioxidant activity

#### Estimation of catalase

Catalase activity was estimated by the method of Hugo E Aebi (1974). To 1 ml phosphate buffer (50 mM, pH 7.0), 100 μl of supernatant was added followed by the addition of 0.5 ml of freshly prepared hydrogen peroxide (30 mM; Sigma Aldrich). After adding 2 ml dichromate acetic acid solution (HiMedia), the tubes were placed for 10 min in a boiling water bath and was read at 240 nm by calculation (Decrease in absorbance × 100/1) divided by protein amount in mg divided by time in min = units/mg protein/min.

#### Estimation of superoxide dismutase

Superoxide dismutase was estimated by the method of Paoletti and Mocali (1990) by assessing the inhibition of NADH oxidation using β-mercaptoethanol (Merck) in the presence of EDTA (Sigma Aldrich) and Mn as a substrate. The assays were run by adding to the cuvette sequentially 0.80 ml of 50 mM phosphate buffer (pH 7.4), 55 μl EDTA/Mn solution of 100/50 mM, 40 μl NADH solution (Sigma Aldrich) of 7.5 mM, and different volumes of tissue extract. The reaction was then initiated by adding 100 μl 10 mM β-mercaptoethanol solution. The change in the absorbance of NADH at 340 nm per min was noted followed by calculating the enzyme activity from the calibration curve.

#### Estimation of glutathione peroxidase and reduced glutathione

GPx activity was measured using 5, 5, dithiobis-tetranitrobenzoic acid (DTNB) reagent (Sigma Aldrich), according to Hafeman et al. (1974). Briefly, the samples were incubated at 37 °C for 5 min with a solution containing 80 mM sodium phosphate buffer (pH 7.0), 80 mM EDTA, 1 mM sodium azide (NaN_3;_HiMedia), 0.4 mM GSH, and 0.25 mM hydrogen peroxide (reagent solution). The reaction was stopped by adding metaphosphoric acid solution for protein precipitation. After centrifugation, the remaining GSH in the supernatant was determined using 0.4 M sodium phosphate buffer (pH 7.0) and 1 mM DTNB in 1% trisodium citrate solution, and then it was measured at 412 nm according to Boyne and Ellman (1972) where the enzyme activity is expressed in terms of μg of glutathione utilized/min/mg protein.

#### Estimation of glutathione reductase

Glutathione reductase was measured according to the method of Goldberg and Spooner (1983). To 0.2 ml of the sample, 1.5 ml of phosphate buffer, 0.5 ml of EDTA, 0.2 ml of oxidized glutathione (HiMedia) and 0.1 ml NADPH (HiMedia) were added. The decrease in the optical density of the enzyme was measured against that of the blank at 340 nm. The enzyme activity has been expressed as nM NADPH oxidized to NADP/mg of protein/min by using the molar extinction coefficient of 6200/M/cm at 340 nm.

### Innate immunological response

#### Lysozyme assay

Lysozyme activity was determined by the protocol described by Parry et al. (1965). 25 μl of serum was added to 175 μl of *M. lysodiekticus* (Sigma Aldrich) suspension 0.3 mg ml^−1^ in 0.1 M phosphate citrate buffer, pH 5.8. After a rapid mixing, the change in turbidity was measured every 30 s for 5 min at 540 nm at approximately 25 °C using a micro-plate reader. The equivalent unit of the activity of the sample (compared with the standard) were determined and expressed in μg ml^−1^ serum. Delta T% (*y*) = [Abs (0 s) – Abs (300 s)] × 100.

#### Respiratory burst activity

Respiratory burst activity was carried out as described by Anderson and Siwicki (1995) for measuring the production of oxygen radicals from phagocytes. 0.1 ml of heparinized blood was placed into a microtiter plate and equal amount of 0.2% NBT (Merck) was added and incubated for 30 min at room temperature. To a glass tube containing 1.0 ml of N, N-dimethylformamide solution (Merck), 0.05 ml of the NBT-blood cell suspension was added. Then the mixture was centrifuged at 3000 g for 5 min. The absorbance of the supernatant taken into a glass cuvette was read at 540 nm using a spectrophotometer. Spontaneous O_2_^−^ production = (Abs NBT reduction of sample) − (Abs of blank).

#### Myeloperoxidase activity

Peroxidase activity was determined following the methods of Quade and Roth (1997). 5 μL of serum was placed in a flat bottomed 96-well plate in triplicates. Thereafter, 45 μL of Hank’s balanced salt solution (HBSS; Merck) without Ca^2+^ or Mg^2+^ and 100 μL of solution (40 ml of distilled water, 10 μL of H2O2 (30%—Sigma-Aldrich), 1 pill of 3, 3′, 5, 5′-tetramethylbenzidine (TMB; Sigma Aldrich) were added. 50 μL 2 M H_2_SO_4_ was added immediately once the colour change appeared and the optical density was read at 450 nm in a plate reader. One unit was defined as the amount producing an absorbance change of 1 and the activity was expressed as units (U) mg^−1^ serum or mucus proteins.

## Histology

For histopathology study of gills and liver, tissues were processed following the methods described by Humason (1979) and Pearse (1968). Gills and liver were fixed for 24 h in 10% formalin solution before embedding in paraffin wax. Sections of the liver and gills were stained with haematoxylin and eosin and were analysed for histopathological alterations.

## Inductively coupled plasma mass spectrometry

Gills, liver and muscle samples were prepared for ICP-MS as described by Shahzad et al. (2017). 10 ml concentrated nitric acid and 2 ml of perchloric acid were added to 1 g of freeze-dried samples which were digested by heating in a hot plate at 100 °C. Two drops of hydrogen peroxide were added to the samples which were then diluted with distilled water and finally were filtered with a Whatman filter paper.

## Determination of DNA damage marker 8-hydroxy-2-deoxyguanosine

8-Hydroxy-2-deoxyguanosine (8-OHdG), a biomarker of oxidative DNA damage was measured with the highly sensitive 8-OHdG competitive ELISA kit (Cellbiolabs, USA). For the quantification of 8-OHdG, blood plasma was collected from the blood of four fish by centrifugation for 5 min at 5000*g* and stored at −18^°^ until analysis.

## Statistical analysis

One-way ANOVA and Duncan’s multiple range test (DMRT) were used to determine the significant differences between the means using SPSS version 16 for windows. Mean values were considered significantly different when *P* < 0.05. Data are presented as means ± standard deviation. Histology results were not statistically analysed, but visually examined.

## Results

### Characterization of zinc oxide nanoparticles

**Fig. 1:**
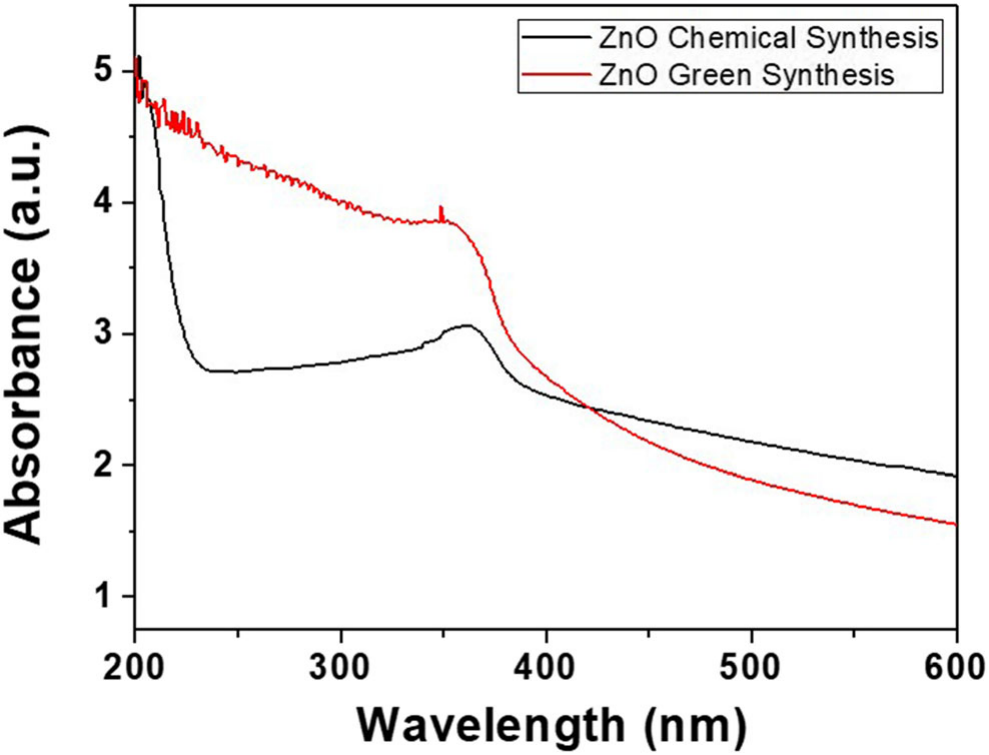
**a** UV-Vis spectrum of chemically synthesized ZnO NPs. **b** UV-Vis spectrum of green synthesized ZnO NPs

The colour of the solution for chemically synthesized ZnO NPs was observed to be white, whereas the colour of green synthesized ZnO NPs showed a pale white colour. The UV-Vis absorption spectrum of chemically synthesized ZnO NPs exhibited an absorption wavelength of 360 nm (Fig. 1) and that of green synthesized ZnO NPs was 350 nm (Fig. 1).

**Fig. 2:**
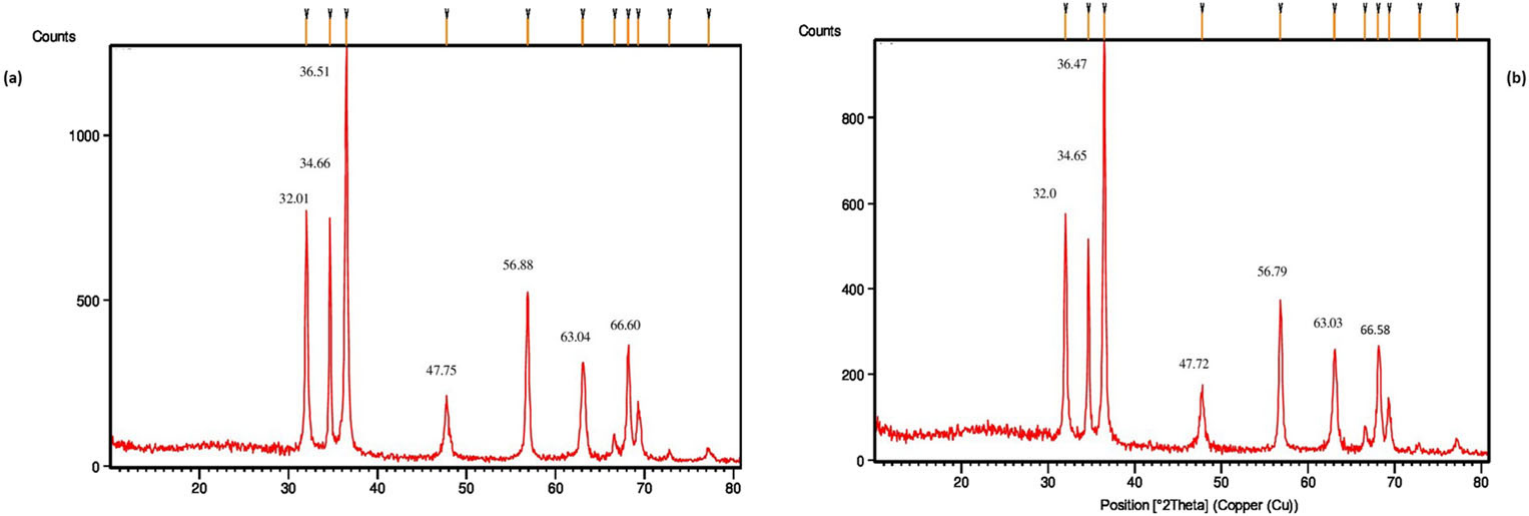
**a** XRD pattern of chemically synthesized ZnO NPs. **b** XRD pattern of green synthesized ZnO NPs

X-ray diffraction pattern (XRD) of chemically synthesized ZnO NPs showed 2 *θ* values at 32.01° (100), 34.66° (002), 36.51° (101), 47.75° (102), 56.88° (110), 63.04° (103) and 66.60° (200) and that of green synthesized ZnO NPs exhibited 2 *θ* values at 32.0° (100), 34.65° (002), 36.47° (101), 47.72° (102), 56.79° (110), 63.03° (103) and 66.58° (200) corresponding with the Joint Committee on Powder Diffraction Standard (JCPDS) Card No. 89-1397 (Fig. 2a, b). The peaks observed were the indication of the wurtzite structure of zinc oxide. The average crystallite size of chemically synthesized ZnO NPs and green synthesized ZnO NPs were calculated as 29.5 nm and 35.10 nm respectively as calculated by the Scherrer equation.

**Fig. 3:**
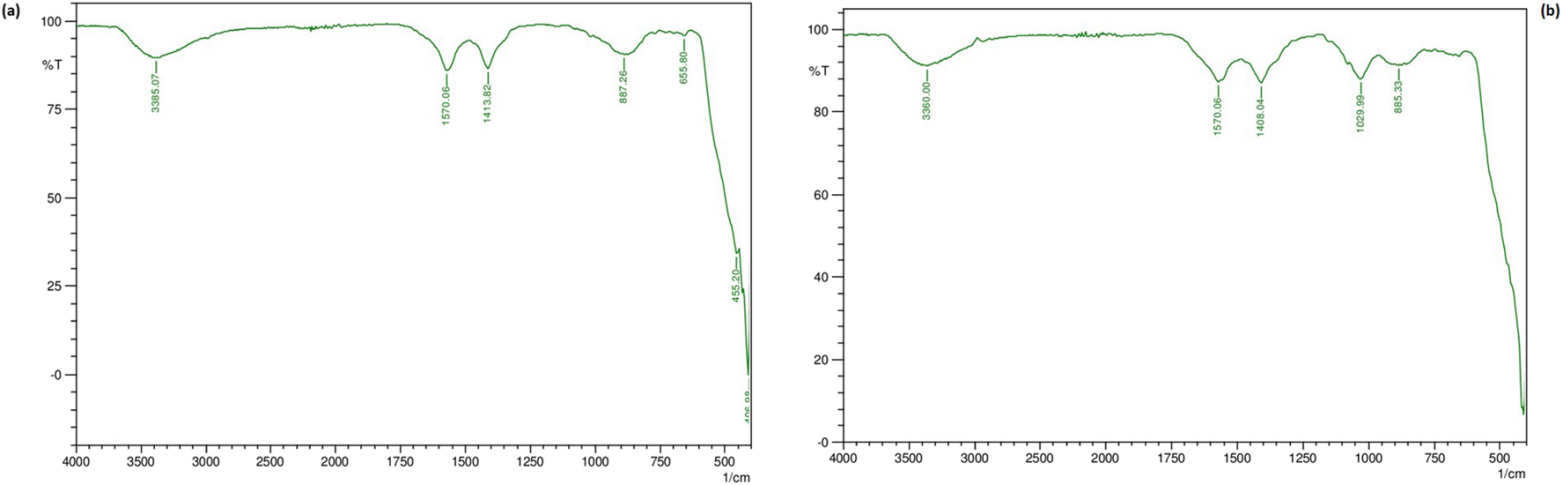
**a** FTIR spectra of chemically synthesized ZnO NPs. **b** FTIR spectra of green synthesized ZnO NPs

Figure 3 a and b show the FTIR absorption spectrum of chemically synthesized ZnO NPs and green synthesized ZnO NPs respectively. The peak at 455.20 cm^−1^ indicates absorption of ZnO bond and the broad absorption peak at 3385.07 cm^−1^ can be attributed to the absorption of hydroxyl (Fig. 3a). In the spectra of green synthesized ZnO NPs, the peak at 885.33 cm^−1^ is probably linked to ZnO whereas the broad absorption peak at 3360 cm^−1^ can be assigned to hydrogen bonded in alcohol or phenol groups (Fig. 3b).

**Fig. 4.**
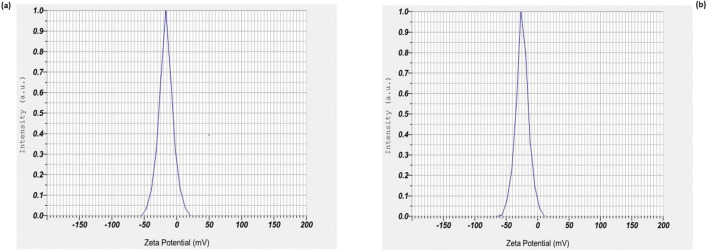
**a** Zeta potential of chemically synthesized ZnO NPs. **b** Zeta potential of green synthesized ZnO NPs

**Fig. 5:**
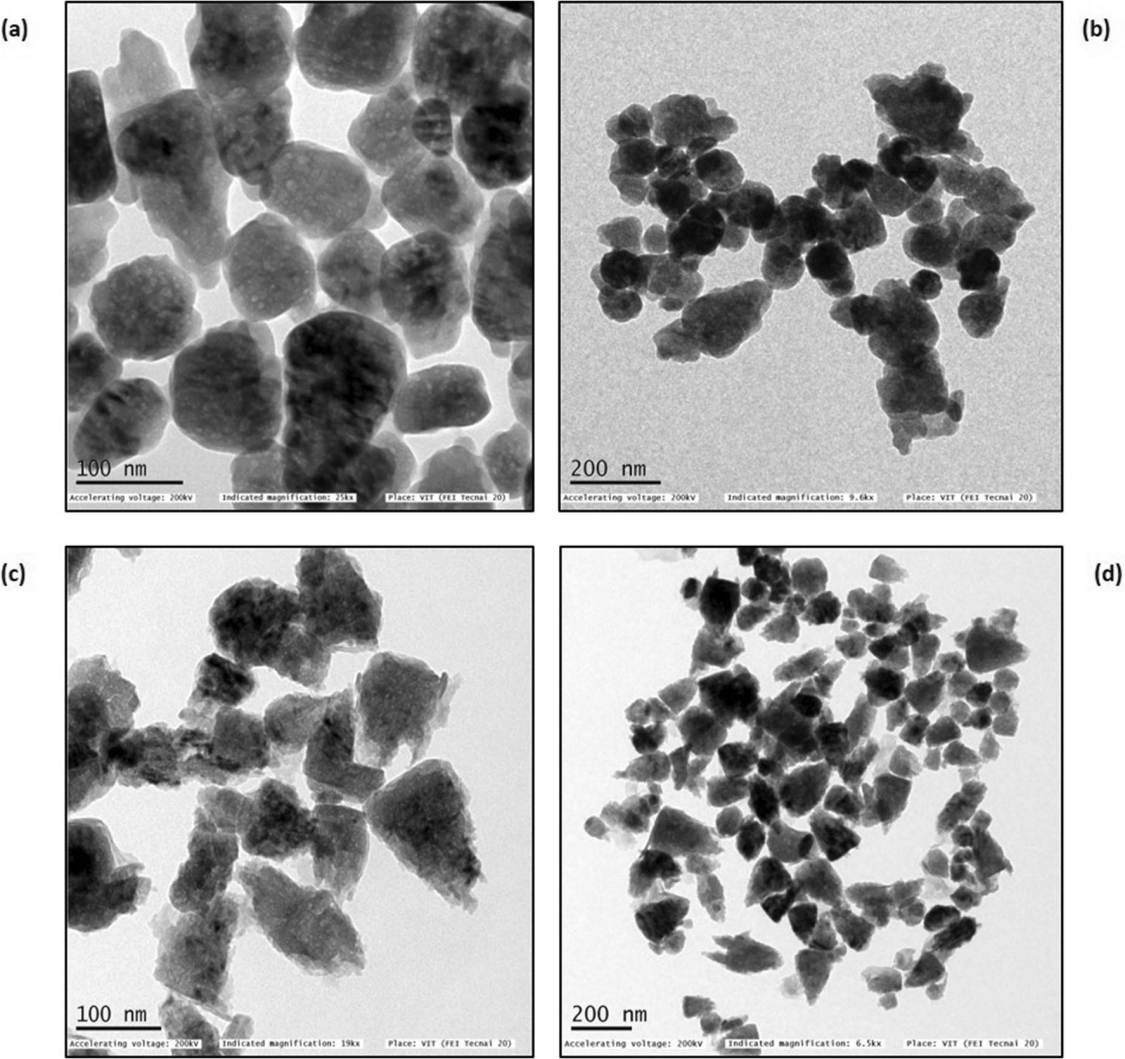
**a**, **b** TEM images of chemically synthesized ZnO NP at 100 nm and 200 nm respectively. **c**, **d** TEM images of green synthesized ZnO NP at 100 nm and 200 nm respectively

Zeta potential of chemically synthesized ZnO NPs and green synthesized ZnO NPs were determined in water as a dispersant. The measurement demonstrated that chemically synthesized ZnO NPs have a zeta potential of − 16.6 mV (Fig. 4a) and that of green synthesized ZnO NPs have a zeta potential of − 24.5 mV (Fig. 4b). TEM images determined the morphological features of nanoparticles (Fig. 5a–d). The TEM image of green synthesized nanoparticles shows mostly spherical shapes with a few rod shapes, whereas the chemically synthesized nanoparticles display agglomeration of different shaped particles such as triangles, rods, spheres and hexagonal shapes.

### In vitro antioxidant activity

**Fig. 6:**
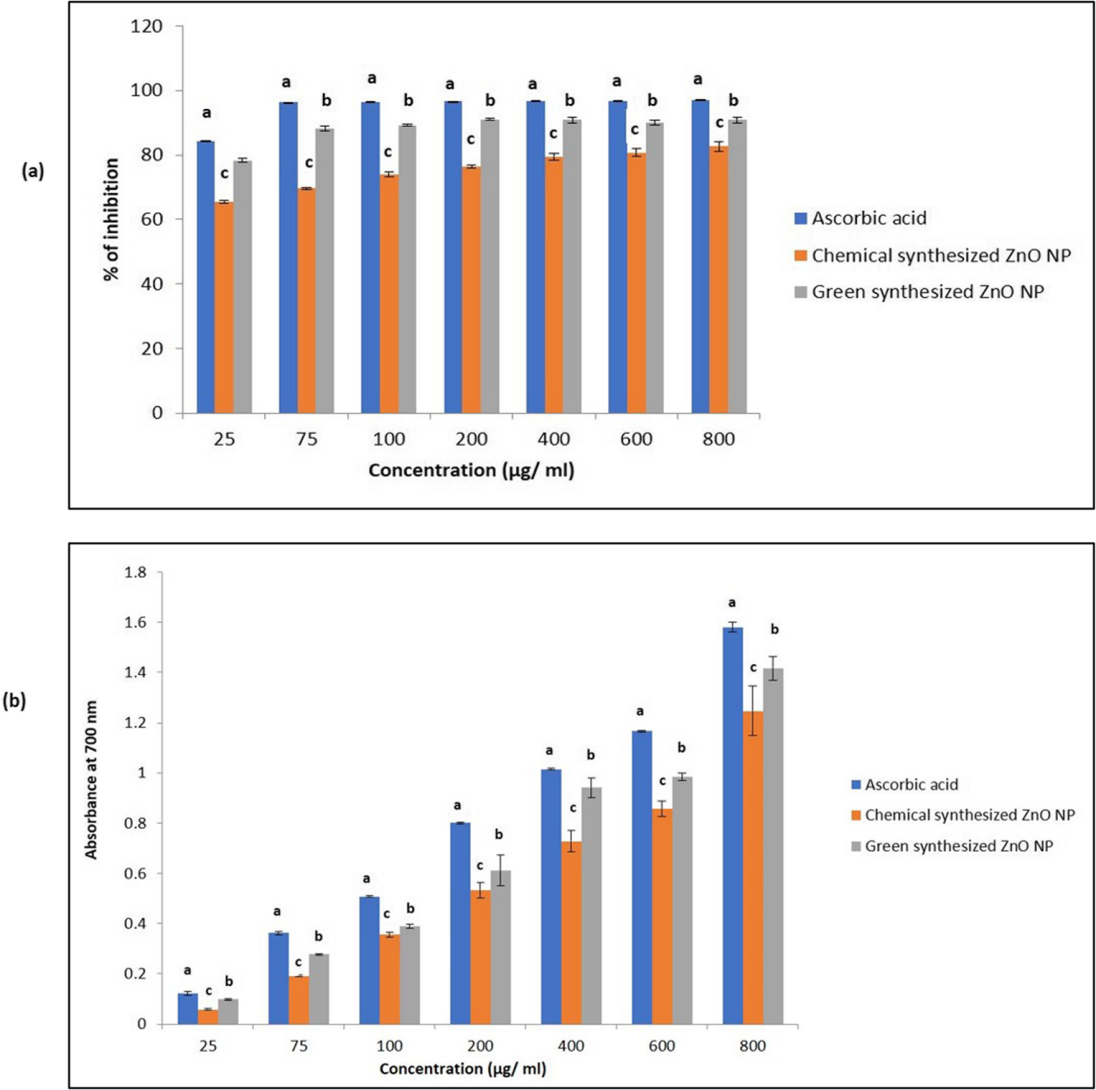
**a** DPPH activity of chemically synthesized and green synthesized ZnO NP. Bars assigned with different superscripts are significantly different (*P* < 0.05). **b** Reducing power activity of chemically synthesized and green synthesized ZnO NP. Bars assigned with different superscripts are significantly different (*P* < 0.05)

Antioxidant capacity of chemically synthesized ZnO NPs and green synthesized ZnO NPs were estimated by DPPH assay and reducing power assay. In DPPH assay, the antioxidant activity of chemically synthesized ZnO NPs and green synthesized ZnO NPs improved as the concentration of samples increased from 25 to 800 μg/ml (Fig. 6a) and a colour change was observed from deep violet to pale yellow. It was observed that the free radical scavenging activity of green synthesized ZnO NPs increased in a concentration-dependent manner. Also, it was observed that the antioxidant potential of green synthesized ZnO NPs was significant to chemically synthesized ZnO NPs. Thus, green synthesized ZnO NPs exhibited better antioxidant property than chemically synthesized ZnO NPs.

In reducing power assay, a change in colour was observed at 700 nm from yellow to shades of blue or green. As per the results, the reducing activity of green synthesized ZnO NPs was higher than chemically synthesized ZnO NPs. Reducing activity was observed to be increasing as the concentration increased (Fig. 6b).

### Antibacterial activity

**Fig. 7:**
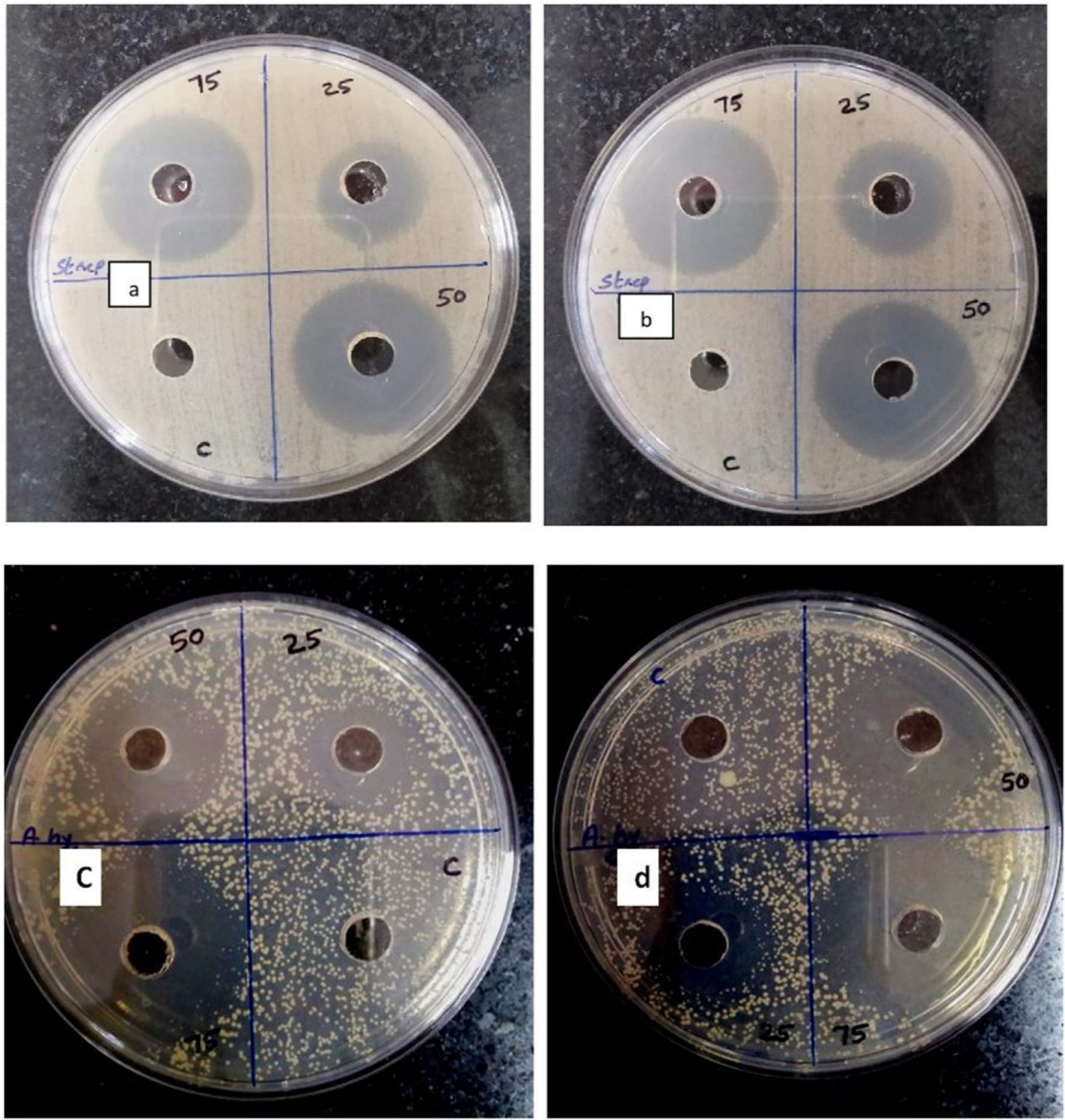
**a** Zone of inhibition observed in chemically synthesized ZnO NP plates tested against *Streptococcus agalactiae* at 25-, 50- and 75-μg/ml concentrations. **b** Zone of inhibition observed in green synthesized ZnO NP plates tested against *Streptococcus agalactiae* at 25-, 50- and 75-μg/ml concentrations. **c** Zone of inhibition observed in chemically synthesized ZnO NP plates tested against *Aeromonas hydrophila* at 25-, 50- and 75-μg/ml concentrations. **d** Zone of inhibition observed in green synthesized ZnO NP plates tested against *Aeromonas hydrophila* at 25-, 50- and 75-μg/ml concentrations

The antibacterial activity of green synthesized and chemically synthesized ZnO NPs were investigated against gram-negative *A. hydrophila* and gram-positive *S. agalactiae* with three different concentrations (25, 50 and 75 μg/ml) by Kirby-Bauer disc diffusion method (Fig. 7a–d). Among the tested pathogens, *A. hydrophila* exhibited higher sensitivity toward green synthesized ZnO NPs with 35 mm zone of inhibition at the highest concentration (75 μg/ml) while the zone of inhibition at the highest concentration of chemically synthesized ZnO NPs was 32 mm. On the other hand, *S. agalactiae* too exhibited higher sensitivity to green synthesized ZnO NPs at the highest concentration with a 31-mm zone of inhibition and the zone of inhibition was 29 mm in the plate of chemically synthesized ZnO NPs. This implies that both gram-positive and gram-negative bacteria seemed to have less resistance to green synthesized ZnO NPs when compared with chemically synthesized ZnO NPs.

### Enzymatic and non-enzymatic antioxidant enzymes

**Fig. 8:**
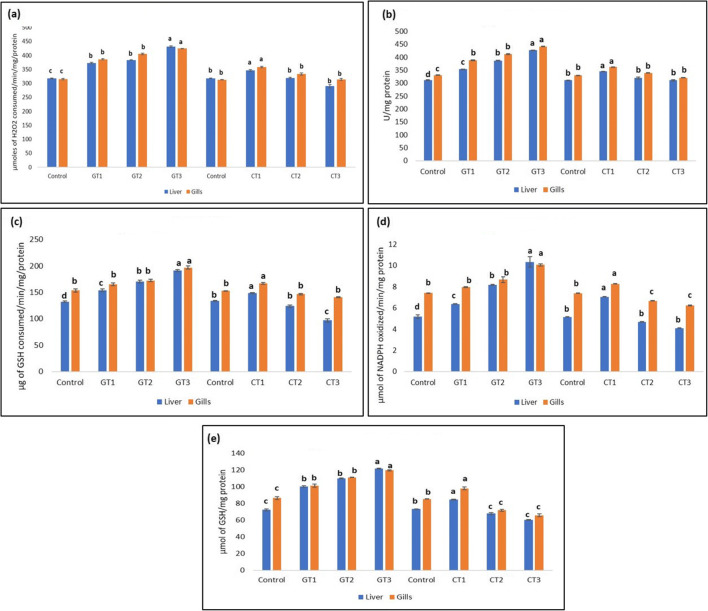
**a** Activity of catalase (CAT) enzyme in the liver and gills in the control group and in GT1 –group fed green synthesized ZnO NPs at concentration 100 mg/kg, CT1—fish fed chemically synthesized ZnO NPs at concentration 100 mg/kg, GT2—fish fed green synthesized ZnO NPs at concentration 200 mg/kg, CT2—fish fed chemically synthesized ZnO NPs at concentration 200 mg/kg, GT3—fish fed green synthesized ZnO NPs at concentration 400 mg/kg, CT3—fish fed chemically synthesized ZnO NPs at concentration 400 mg/kg. Values are expressed as mean ± SD (*n* = 4). Bars assigned with different superscripts are significantly different (*P* < 0.05). **b** Activity of superoxide dismutase (SOD) enzyme in the liver and gills in the control group and in GT1—fish fed green synthesized ZnO NPs at concentration 100 mg/kg, CT1—fish fed chemically synthesized ZnO NPs at concentration 100 mg/kg, GT2—fish fed green synthesized ZnO NPs at concentration 200 mg/kg, CT2—fish fed chemically synthesized ZnO NPs at concentration 200 mg/kg, GT3—fish fed green synthesized ZnO NPs at concentration 400 mg/kg, CT3—fish fed chemically synthesized ZnO NPs at concentration 400 mg/kg. Values are expressed as mean ± SD (*n* = 4). Bars assigned with different superscripts are significantly different (*P* < 0.05). **c** Activity of glutathione peroxidase (GPx) enzyme in the liver and gills in the control group and in GT1—fish fed green synthesized ZnO NPs at concentration 100 mg/kg, CT1—fish fed chemically synthesized ZnO NPs at concentration 100 mg/kg, GT2—fish fed green synthesized ZnO NPs at concentration 200 mg/kg, CT2—fish fed chemically synthesized ZnO NPs at concentration 200 mg/kg, GT3—fish fed green synthesized ZnO NPs at concentration 400 mg/kg, CT3—fish fed chemically synthesized ZnO NPs at concentration 400 mg/kg. Values are expressed as mean ± SD (*n* = 4). Bars assigned with different superscripts are significantly different (*P* < 0.05). **d** Activity of reduced glutathione (GSH) enzyme in the liver and gills in the control group and in GT1—fish fed green synthesized ZnO NPs at concentration 100 mg/kg, CT1—fish fed chemically synthesized ZnO NPs at concentration 100 mg/kg, GT2—fish fed green synthesized ZnO NPs at concentration 200 mg/kg, CT2—fish fed chemically synthesized ZnO NPs at concentration 200 mg/kg, GT3—fish fed green synthesized ZnO NPs at concentration 400 mg/kg, CT3—fish fed chemically synthesized ZnO NPs at concentration 400 mg/kg. Values are expressed as mean ± SD (*n* = 4). Bars assigned with different superscripts are significantly different (*P* < 0.05). **e** Activity of glutathione reductase (GR) enzyme in the liver and gills in the control group and in GT1—fish fed green synthesized ZnO NPs at concentration 100 mg/kg, CT1—fish fed chemically synthesized ZnO NPs at concentration 100 mg/kg, GT2—fish fed green synthesized ZnO NPs at concentration 200 mg/kg, CT2—fish fed chemically synthesized ZnO NPs at concentration 200 mg/kg, GT3—fish fed green synthesized ZnO NPs at concentration 400 mg/kg, CT3—fish fed chemically synthesized ZnO NPs at concentration 400 mg/kg. Values are expressed as mean ± SD (*n* = 4). Bars assigned with different superscripts are significantly different (*P* < 0.05)

Fish that were fed green synthesized ZnO NPs at all concentrations (100 mg/kg, 200 mg/kg and 400 mg/kg) significantly (*P* < 0.05) enhanced catalase (CAT) activity, glutathione peroxidase (GPx) and superoxide dismutase (SOD) in the liver and gills in a concentration dependent manner. On the other hand, chemically synthesized ZnO NPs at all three concentrations (100 mg/kg, 200 mg/kg and 400 mg/kg) inhibited the activity of CAT, GPx and SOD in the liver and gills (Fig. 8a–e).

All concentrations of green synthesized ZnO NP feeds (GT1, GT2 and GT3) significantly (*P* < 0.05) augmented the activity of glutathione reductase (GR) and reduced glutathione (GSH) in the gills and liver as the concentration increased; however, fish fed chemically synthesized ZnO NP feed induced a significant decline in the activity of GR and GSH in both the liver and gills with the least activity observed in CT3.

### Innate immunological response

#### Lysozyme activity

**Fig. 9:**
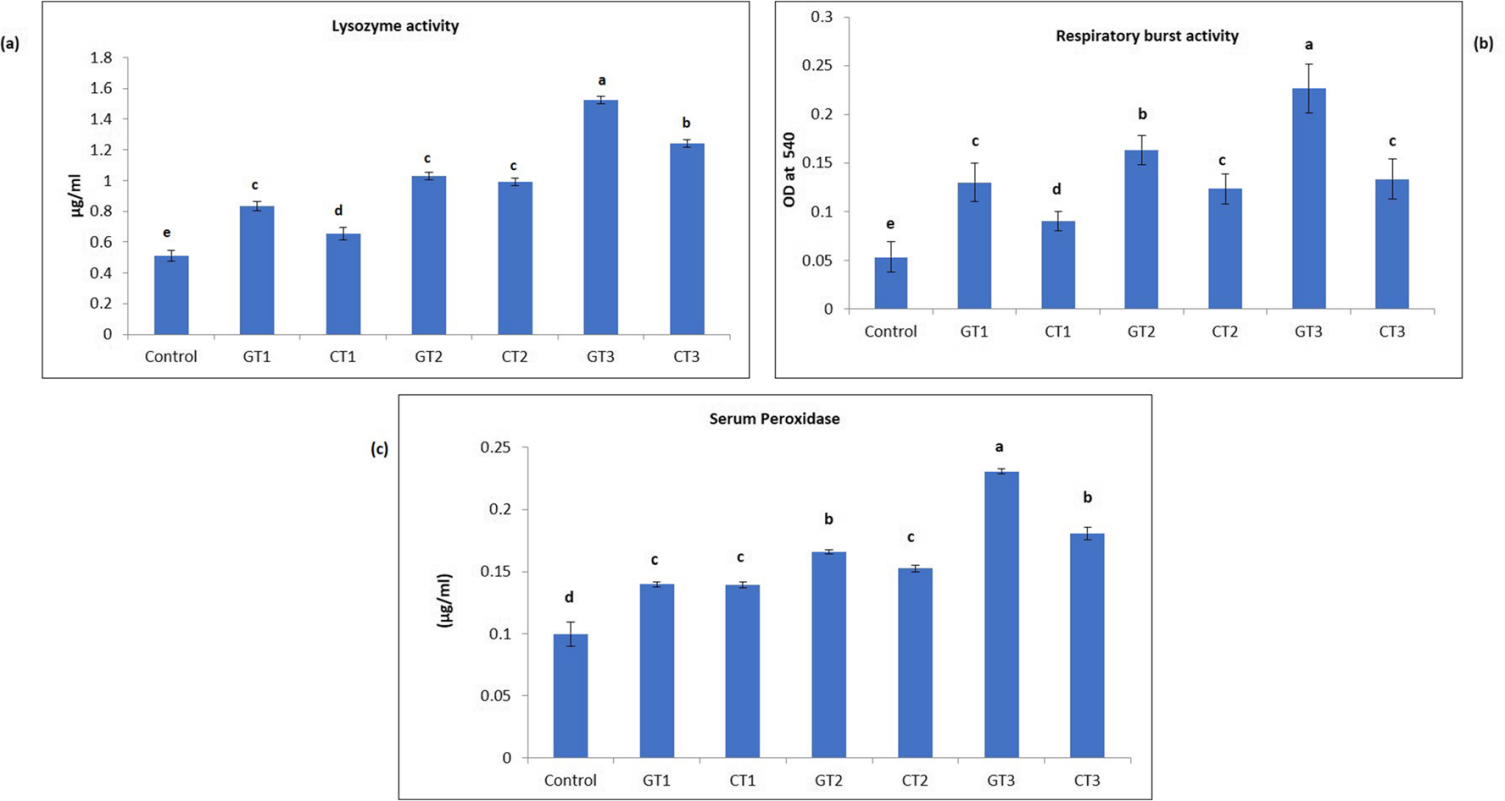
**a** Lysozyme activity in the control group and in GT1—group fed green synthesized ZnO NPs at concentration 100 mg/kg, CT1—group fed chemically synthesized ZnO NPs at concentration 100 mg/kg, GT2—group fed green synthesized ZnO NPs at concentration 200 mg/kg, CT2—group fed chemically synthesized ZnO NPs at concentration 200 mg/kg, GT3—group fed green synthesized ZnO NPs at concentration 400 mg/kg, CT3—group fed chemically synthesized ZnO NPs at concentration 400 mg/kg. Values are expressed as mean ± SD (*n* = 4). Bars assigned with different superscripts are significantly different (*P* < 0.05). **b** Respiratory burst activity in the control group and in GT1—group fed green synthesized ZnO NPs at concentration 100 mg/kg, CT1—group fed chemically synthesized ZnO NPs at concentration 100 mg/kg, GT2—group fed green synthesized ZnO NPs at concentration 200 mg/kg, CT2—group fed chemically synthesized ZnO NPs at concentration 200 mg/kg, GT3—group fed green synthesized ZnO NPs at concentration 400 mg/kg, CT3—group fed chemically synthesized ZnO NPs at concentration 400 mg/kg. Values are expressed as mean ± SD (*n* = 4). Bars assigned with different superscripts are significantly different (*P* < 0.05). **c** Serum peroxidase activity the control group and in GT1—group fed green synthesized ZnO NPs at concentration 100 mg/kg, CT1—fish fed chemically synthesized ZnO NPs at concentration 100 mg/kg, GT2—fish fed green synthesized ZnO NPs at concentration 200 mg/kg, CT2—fish fed chemically synthesized ZnO NPs at concentration 200 mg/kg, GT3—fish fed green synthesized ZnO NPs at concentration 400 mg/kg, CT3—fish fed chemically synthesized ZnO NPs at concentration 400 mg/kg. Values are expressed as mean ± SD (*n* = 4). Bars assigned with different superscripts are significantly different (*P* < 0.05)

Results of lysozyme activity revealed that green synthesized ZnO NP feeds and chemically synthesized ZnO NP feeds significantly (*P* < 0.05) increased lysozyme activity in Nile tilapia in a concentration-dependent manner when compared with the control (Fig. 9a). Maximum activity was detected in fish that were fed GT3 diet.

#### Respiratory burst activity

It was observed that all treatment groups exhibited significantly higher (*P* < 0.05) respiratory burst activity when compared with the control (Fig. 9b). Among all the treatment groups, maximum activity was observed in the fish that were fed GT3 diet.

#### Myeloperoxidase activity

Serum peroxidase activity was observed to be significantly higher (*P* < 0.05) in all treatment groups when compared with the control (Fig. 9c). Fish that were fed diet GT3 has the maximum peroxidase activity in the serum.

### Histology

**Fig. 10:**
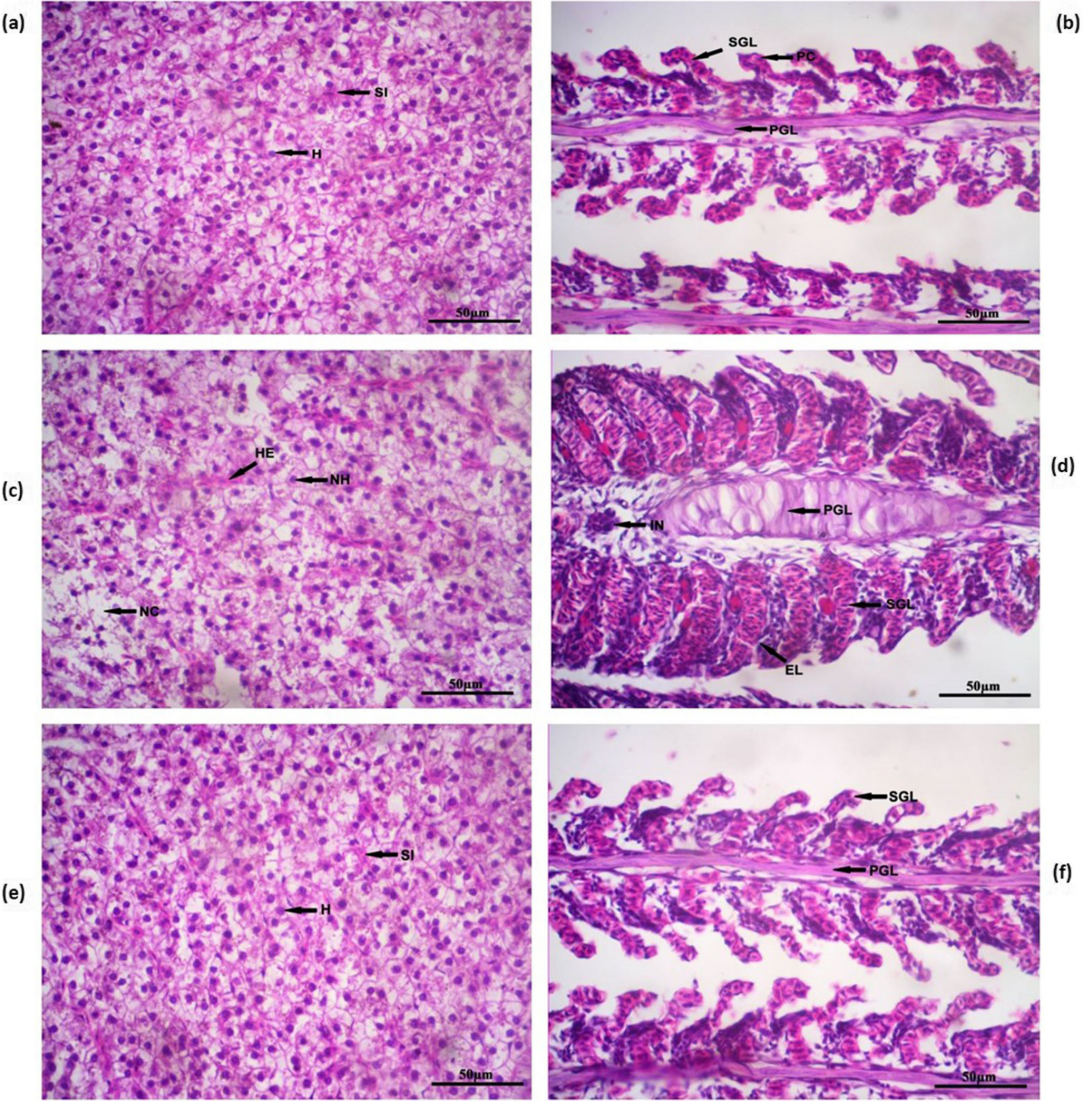
**a** Histology of liver section of Nile tilapia in the control group showing normal architectural arrangement with abundant hepatocytes (H) with prominent nucleus and sinusoids (SI). **b** Histology of gill section of Nile tilapia in the control group showing secondary gill lamellae (SGL), primary gill lamella (PGL), a single layer of epithelial cells supported by the pillar cells (PC). **c** Histology of liver section of Nile tilapia in the group fed chemically synthesized ZnO NP–enriched diet showing hepatocytes with moderate necrosis (NH), some area showing amorphous aggregates of necrotic material (NC) and moderate congestion in blood sinusoids (HE). **d** Histology of gill section of Nile tilapia in the group fed chemically synthesized ZnO NP–enriched diet showing moderate epithelial hyperplasia, epithelial lifting (EL) and inflammation (IN). **e** Histology of liver section of Nile tilapia in the group fed green synthesized ZnO NP–enriched diet showing normal histology with abundant hepatocytes (H) with prominent nucleus and sinusoids (SI). **f** Histology of gill section of Nile tilapia in the group fed green synthesized ZnO NP–enriched diet showing normal histology with secondary gill lamellae (SGL) and primary gill lamella (PGL)

Effects of dietary intake of ZnO NP feeds on gills and liver histology of control and treatment groups are shown in Fig. 10a–f. In the control group, histological analysis of the liver revealed normal architectural arrangement with abundant hepatocytes (H) with prominent nucleus and sinusoids (SI). Gills of the control group also displayed normal structure with secondary gill lamellae (SGL), primary gill lamella (PGL), a single layer of epithelial cells supported by the pillar cells (PC). In the fish fed chemically synthesized ZnO NP feed, hepatocytes of the liver were observed to have moderate necrosis (NH), with some areas showing amorphous aggregates of necrotic material (NC) and moderate congestion in blood sinusoids (HE). On the other hand, the histopathology of gills of fish fed chemically synthesized ZnO NP feed reported moderate epithelial hyperplasia, epithelial lifting (EL) and inflammation (IN). But in the case of green synthesized ZnO NP–fed fish, both the liver and gills revealed normal histology.

### Inductively coupled plasma mass spectrometry

The accumulation of zinc from ZnO NP feed was determined by ICP-MS analysis. Liver, gill and muscle samples of fish fed with the highest dose (CT3 and GT3) were selected for the analysis. From the studied tissues, the order of accumulation of zinc was liver > muscle > gills in fish fed both diets. Significantly higher (*P* < 0.05) accumulation of zinc was detected in all the three tissues of fish fed CT3 and GT3 when compared with the control (Table 1). The highest Zn accumulation was observed in the liver at a higher dose of feed, and the mean value of Zn in the liver was 133.17 ± 0.37 ppm in CT3 diet–fed fish and 113.03 ± 0.11 ppm in GT3 diet–fed fish. The least accumulation of Zn was reported in the gill tissues 57.11 ± 0.19 ppm in the CT3 diet–fed fish and 37.92 ± 0.31 ppm in GT3 diet–fed fish. Muscle tissues were reported to have Zn content less than that in the liver with 89.91 ± 0.64 ppm in CT3 diet–fed fish and 59.12 ± 0.62 ppm in GT3 diet–fed fish. In all the three organs tested, fish fed CT3 diet reported to have the higher Zn content than the fish fed GT3 diet.

**Table 1:** Zn (ppm) accumulation from ZnO NPs in the liver, gills and muscles of Nile tilapia. Values are expressed as mean ± SD (*n* = 4). Values in a row with different superscripts are significantly different (*P* < 0.05)

Tissues	Control-Zn content (ppm/g tissue)	CT3 NP-Zn content (ppm/g tissue)	GT3 NP-Zn content (ppm/g tissue)
Liver	0.152 ± 0.001^c^	133.17 ± 0.37 ^a^	113.03 ± 0.11^b^
Gills	0.053 ± 0.001^c^	57.11 ± 0.19 ^a^	37.92 ± 0.31 ^b^
Muscle	0.043 ± 0.001^c^	89.91 ± 0.64^a^	59.12 ± 0.62 ^b^

### Determination of oxidative damage in DNA

Results of the measurement of 8-OHdG levels suggest that DNA damage is significantly (*P* < 0.05) higher in fish fed chemically synthesized ZnO NP–enriched feed (CT1, CT2, CT3) when compared with the control. However, the DNA damage was found to be less in the plasma of fish fed green synthesized ZnO NP incorporated feed (Table 2).

**Table 2:** Plasma 8-OHdG levels in Nile tilapia. Values are expressed as mean ± SD (*n* = 4). Values in a row with different superscripts are significantly different (*P* < 0.05)

	Control	CT1	CT2	CT3	Control	GT1	GT2	GT3
Plasma 8-OHdG levels (ng/ml)	2.9 ± 0.006^d^	3.2 ± 0.002^c^	3.5 ± 0.003^b^	3.89 ± 0.008^a^	2.90 ± 0.003^a^	2.64 ± 0.008^b^	2.35 ± 0.002^c^	1.99 ± 0.003^d^

## Discussion

The present study evaluated the possible effects of dietary ZnO NPs prepared by green and chemical methods on various health parameters in Nile tilapia. First, the structural properties of nanoparticles were confirmed by beginning with Uv-Vis spectrum analysis. UV-Vis spectrum of chemically synthesized and green synthesized ZnO NPs are shown in Fig. 1. Due to the surface plasmon absorbance of ZnO NPs, absorption peaks were observed at 360 nm for chemically synthesized ZnO NPs and 350 nm for green synthesized ZnO NPs which confirmed the formation of zinc oxide nanoparticles. Similar results have been documented by Jayarambabu et al. (2017) and Chaudhuri and Malodia (2017). Various Bragg’s reflection peaks were revealed in the XRD spectrum and the absence of a considerable shift in the diffraction peaks of chemical and green synthesized ZnO NPs indicate the absence of crystalline impurities, the results of which correlates with the previously reported findings, where Jamdagni et al. (2018) synthesized zinc oxide nanoparticles using flower extract of *Nyctanthes arbortristis* whereas, Mahendiran et al. (2017) compared ZnO NPs synthesized using *Aloe vera* and *Hibiscus sabdariffa* with the chemical method.

Next was FTIR analysis that was performed to determine the functional groups responsible for capping and stabilization of ZnO NPs in the extract of *L. aspera* and oxy-cyclodextrin complex. Alcohols, phenols and various other phytochemicals in *L. aspera* interact with the zinc surface and help in the stabilization of ZnO NPs. Our results match with the already reported results where ZnO NPs were green synthesized using extracts of *Calotropis gigantea* and *Laurus nobilis* (Chaudhuri and Malodia 2017; Fakhari et al. 2019). Zeta potential values of both nanoparticles indicate that they are stable which is due to the electrostatic repulsive force.

Before proceeding to feed preparation, the in vitro antioxidant activity measured by DPPH and reducing power assay revealed that green synthesized ZnO NP solution exhibit higher antioxidant property than chemically synthesized ZnO NPs. However, none of the nanoparticles could exceed the antioxidant property of the standard ascorbic acid in both assays. This result is similar to the already reported result by Safawo et al. (2018).

This was followed by the antibacterial property of nanoparticle solutions and the results of antibacterial activity of ZnO NPs reported that green synthesized ZnO NPs revealed enhanced activity against *A. hydrophila* and *S. agalactiaea*. Previous studies have also confirmed that ZnO NPs exhibits strong antibacterial activity against gram-positive and gram-negative bacteria, also the green synthesized ZnO NPs are reported to have better activity than chemically synthesized ZnO NPs (Vimala et al. 2014; Venkatachalam et al. 2016; Hazra et al. 2013). The possible mechanism of antibacterial activity by ZnO NPs are due to the loss of bacterial cell integrity when ZnO NPs come in direct contact with the cell wall (Zhang et al. 2007), releasing Zn ^2+^ ions that are antimicrobial (Li et al. 2011) and formation of reactive oxygen species (ROS) (Jalal et al. 2010).

Earlier reports have confirmed the involvement of nanoparticles in generating oxidative stress by either inhibiting the antioxidant system of cells (Marisa et al. 2016) or by the production of ROS (Vale et al. 2016) thus inducing a toxic effect. Superoxide dismutase (SOD) and catalase (CAT) are tissue-specific biomarker enzymes that act together to form a first line of defence against oxidative stress where SOD catalyses the transformation of superoxide radical to hydrogen peroxide and CAT catalyses hydrogen peroxide to water and oxygen (Ruas et al. 2008; Cao et al. 2012). When comparing CAT activity in the liver and gills, our results reported a significant increase in CAT activity in the liver and gills of fish fed green synthesized nanoparticle at all concentrations, whereas all other diets enriched with chemically synthesized ZnO NP (CT1, CT2 and CT3) resulted in significant reduction in the activity of CAT in both the liver and gills. Thus, at a higher concentration of green synthesized ZnO NP–enriched feed (GT3), an increase in the antioxidant defence was noticed, which could be due to the increased production of oxygen free radical that boosts the antioxidant activities thus helping the cells to protect from free radical injury (Torres et al. 2002). Similar results were reported in SOD activity in the liver and gills. A previous study has confirmed that more than any other enzyme, the activity of SOD would be affected in the liver, gills and intestine when exposed to nanoparticles (Hao et al. 2009). The increase in CAT activity would be due to the removal of hydrogen peroxide radicals reduced by SOD. The activity of GPx was observed to be significantly higher in the liver and gills of fish fed GT1, GT2 and GT3 diets in a concentration-dependent manner. The rest of the diets exhibited a significant reduction in GPx activity in the liver and gills. The reduction in GPx activity in fish fed with ZnO nanofeed either indicates its decreased potential in breaking hydrogen peroxide or an overproduction of hydrogen peroxide or may be as a result of the action of heavy metals directly on the synthesis of the enzyme. Glutathione peroxidase (GPx) is involved in the detoxification of hydroperoxides where they catalyse the reduction of hydrogen peroxide to water in which reduced glutathione (GSH) acts as the source of hydrogen and gets converted to the oxidized form (Alkaladi 2018). The activity of the GSH enzyme was reported to be significantly declined in the liver and gills of fish fed diets enriched with chemically synthesized ZnO nanopowder. On the other hand, all diets incorporated with green synthesized ZnO nanopowder (GT1, GT2 and GT3) displayed a significant increase in the activity of glutathione reductase (GR) enzyme level in the gills. None of the diets enriched with chemically synthesized ZnO nanopowder could augment the GR activity in the liver and gills. Our results coincide with results already reported, which proved that ZnO NPs inhibited SOD, CAT and GPx activities in juvenile carp (Hao and Chen 2012), and in Nile tilapia where SOD, CAT, GPx, GSH and GR activities were declined (Abdelazim et al. 2018).

Lysozyme, a cationic enzyme that helps in lysing the β-1, 4 glycosidic bonds in the peptidoglycan layer present in the cell wall of the bacterium is considered an important ecotoxicological biomarker in fish (Whyte 2007). Our results report that lysozyme activity in Nile tilapia was increased significantly (*P* < 0.05) in a concentration-dependent manner when fish were fed green synthesized ZnO NP enriched feed and chemically synthesized ZnO NP feed with the maximum lysozyme activity detected in fish that were fed GT3 diet. This suggests that the neutrophils were stimulated to release lysozyme in the presence of nanoparticles. Similarly, dietary zinc oxide and selenium nanoparticle feed were revealed to enhance the lysozyme activity in *Labeo rohita* (Swain et al. 2018), β-1,3 glucan binding protein-based zinc oxide nanoparticle elevated lysozyme activity (Anjugam et al. 2018) which are in line with the present investigation. Only a few studies have been reported so far that determined the lysozyme activity in fish after treating it with nanoparticles. Their results reported diminished activity after 25 days when *Epinephelus coioides* was exposed to copper NPs and copper sulfphate NPs (CuSO_4_) (Wang et al. 2015), in *O. niloticus* after 60 days of exposure to iron oxide nanoparticle (Ates et al. 2016). Another study revealed decreased lysozyme activity in the serum for large ZnO NPs at the lowest concentration on the 14th day (Kaya et al. 2016). Production of oxidative radicals, peroxidase and activation of neutrophils account for the non-specific defence mechanism in fish as oxidative burst or respiratory burst; different cells release reactive oxygen species that are noxious for bacterial pathogens (Semple et al. 2018). In the present study, fishes in all treatment groups exhibited a significantly higher (*P* < 0.05) respiratory burst activity in a concentration-dependent manner when compared with the control, with the maximum activity in the fish that were fed GT3 diet indicating the role of nanoparticle in promoting the activity of macrophages and neutrophils. Myeloperoxidase enzyme exists in the granules of polymorphonuclear neutrophils, macrophage and monocytes of fish. In the present study, serum peroxidase activity was observed to be significantly higher (*P* < 0.05) in all treatment groups when compared with the control with the maximum activity in the serum of fish fed GT3. The results suggest that zinc oxide nanoparticles increased the activity of polymorphonuclear cells.

Cellular changes in the organs due to nanoparticles can be evaluated by histopathology (Shobana et al. 2018). Histological changes were observed in the gills and liver of fish fed chemically synthesized ZnO NP–enriched feed, whereas these organs in the fish fed green synthesized ZnO NP incorporated feed did not show any pathological alterations just like the control sections. This is attributed to the presence of *L. aspera* extract and oxy-cyclodextrin present in the green synthesized nanopowder. Liver section of fish fed diet with chemically synthesized ZnO NP was observed to have moderate necrosis, aggregates of necrotic material also and moderate congestion in blood sinusoids. The liver helps in the detoxification of toxic metabolites, which results in the accumulation of these chemicals in the liver. Evidence from earlier studies where similar alterations in the liver of *O. mossambicus* exposed to ZnO NPs (Shahzad et al. 2018) and exposure to small and large size ZnO NPs for 14 days in the liver of *O. niloticus* (Kaya et al. 2016) support the result of the present study.

Gills are associated with respiratory and osmoregulatory function in fish, hence damage to gills can severely affect these functions and can result in stress (Saber 2011). In the present study, gills of fish fed chemically synthesized ZnO NP feed were observed to have inflammation, epithelial hyperplasia and epithelial lifting. These pathological changes in the gills can help the toxic compounds to have close contact with the vascular system of fish affecting its respiration and eventually the health (Krishnaraj et al. 2016). The results of the present study are supported by the results of gill histopathology in *Cyprinus carpio* exposed to ZnO NP for 10 days (Subashkumar and Selvanayagam 2014). Similarly, aberrations in the gill structure were also observed in *O. mossambicus* exposed to ZnO NP after 14 days (Suganthi et al. 2015).

In the present study, accumulation of Zn varied depending on the type of tissue. The tissue that possessed the most Zn content was the liver of fish fed CT3 and GT3 diet, followed by the muscle and gills. The liver plays the role of chemical assimilation of metals that have entered the body, eventually helping in detoxification (Shahzad et al. 2018). Since the mode of exposure was through diet, Zn that was ingested must have accumulated in the liver. Also, the liver being the site of zinc metabolism has an affinity towards zinc due to the expression of metallothionein, a zinc-binding protein. This results in lesser accumulation of zinc in other organs (Giardina et al. 2009). Results of the present study reported the least Zn content in the gills. Gills help in ion exchange, gas transport and respiratory function (Saber 2011). The gills can be considered the first target organ for toxicants as the gill epithelia are the first to come in contact with the chemicals. Accumulation on gills could be due to the adsorption of Zn in the feed when the feed comes in contact with the water (Hao et al. 2013). Also, the Zn-calcium transport carrier in the gill epithelium helps in the uptake of zinc from water (Giardina et al. 2009). In fish muscle, metal accumulation is usually limited. But in the present study, the tissue with the most Zn content after the liver was the muscle tissue. Even though muscles are not a significant target, in this study, muscles were chosen since it is the edible tissue. Previous studies reported the highest Zn accumulation in the liver of *O. mossambicus* (Shahzad et al. 2018) and in the intestine of *O. niloticus* followed by the liver, gills and muscle (Kaya et al. 2015).

8-Hydroxy-2′-deoxyguanosine (8-OHdG) that is derived from hydroxyl radical attack of deoxyguanosine residues has been widely considered a biomarker of oxidative damage to DNA (Singh et al. 1988). The presence of residual 8-OHdG in DNA results in GC to TA transversion unless repaired prior to DNA replication (Cheng et al. 1992). Formation of 8-OHdG, an oxidatively damaged DNA product, is reported to be accelerated by oxygen radical–producing agents; however, antioxidants and flavonoids help reduce 8-OHdG levels (Oliveira et al. 2010). As mentioned earlier, accumulation of 8-OHdG is promoted by various free radical–producing agents by hydroxylation of deoxyguanosine residues in DNA. Mispairing of damaged guanine is formed due to DNA glycosylase enzyme and is removed as 8-OHdG (Obulesu and Rao 2010). It is assumed that the bioactive compounds present in *L. aspera* and oxy-cyclodextrin helped reduce the factors that form free radicals and may have protected the fish immune system.

Nanoparticles are usually synthesized by conventional methods such as physical and chemical methods and also by the green approach. Conventional methods of nanoparticle synthesis use toxic chemicals, synthetic additives and non-polar solvents which are not safe and eco-friendly. On the other hand, green synthesis of nanoparticles involves bacteria, fungi or plants with antioxidant or reducing properties to develop the desired nanoparticles. This is an eco-friendly approach to synthesize nanoparticles. Among the nanoparticles, ZnO NPs have gained popularity being a versatile semiconductor with excellent stability. However, minimal information is available on the application of ZnO NP in aquaculture. There is no information on the comparative effect of ZnO NPs synthesized by the chemical method and by the green approach on the overall health status of fish. This study aimed at analysing the possible toxic effect and changes in the health of Nile tilapia by feeding diets incorporated with chemically synthesized and green synthesized ZnO NP. The various phytochemicals in the *L. aspera* extract acted as capping and stabilizing agent in the green synthesis, whereas the oxy-CD complex has encapsulation efficiency with antioxidant property. The combined action of these two plant-based extracts might have improved the antioxidant ability of green synthesized ZnO NP. This was proven to be true when the present study compared the changes induced by both types of NP through dietary administration in Nile tilapia. There is great importance for this study. To the best of our knowledge, this is the first record about the comparative effects of dietary nanozinc that was prepared by the green method and conventional chemical method in Nile tilapia. In short, the observations from this study conclude that green synthesized ZnO NPs prepared by combining two plant-derived extracts at the highest dose (400 mg/kg) in the diet can ameliorate the activity of antioxidant enzymes thereby reducing oxidative stress to an extent and improving the immune status without inducing any histological alterations in the organs of fish. Further studies to analyse the transcriptomic profile changes and extensive field trials are needed to improve our understanding of the benefits of nanozinc feed further.

## Data Availability

The datasets used and/or analysed during the current study are available from the corresponding author on reasonable request.
